# Research in Mental Health During the Pandemic

**DOI:** 10.1192/j.eurpsy.2022.201

**Published:** 2022-09-01

**Authors:** F. Gaughran

**Affiliations:** South London and Maudsley NHS Foundation Trust, National Psychosis Service, London, United Kingdom

## Abstract

**Abstract Body:**

All healthcare had to rapidly adjust to covid-19; remote options were implemented at pace and unnecessary face to face contact minimised, with infection prevention and control taking primacy. Many research projects were suspended and some clinical researchers moved to frontline care. For psychiatric academic trainees, covid-19 affected recruitment, and risked delaying work on research degrees such as PhDs, potentially beyond the timeframe of a grant, leading to funding uncertainties. Those valuable casual conversations with senior colleagues in the café stopped and with many schools closed, parents had extra pressures on their time at home. In the UK the government prioritised “Urgent Public Health” (UPH) studies and took a co-ordinated approach to research approvals and recruitment strategies, contributing to the success of covid-19 platform trials such as RECOVERY. While initially only a minority of UPH studies were open to people with serious mental illnesses, now the effect of the pandemic on mental health has become a research priority. In parallel, service planners recognised the value of emergent research in informing decision-making creating de facto learning health systems. While covid-19 interrupted research as we knew it, it necessitated new ways of working, some of which will persist. These included an increase in remote data collection, allowing greater access to research opportunities for potential participants, along with more efficient research approval and evidence dissemination pathways.

**Disclosure:**

No significant relationships.

